# Supplementary material for: MetaRibo-Seq measures translation in microbiomes
Source: Nat Commun. 2020 Jun 29;11:3268. doi: 10.1038/s41467-020-17081-z (PMC7324362; doi:10.1038/s41467-020-17081-z)
Supplement: Supplementary file 10 — Supplementary Data 7 [file 41467_2020_17081_MOESM10_ESM.zip › File2/Confidence_High_Taxonomy/223779_out.krona.html]

Javascript must be enabled to view this page.

members
magnitude
magnitudeUnassigned
count
unassigned
taxon
rank

223779\_out

7

2
7
superkingdom

976
7
phylum

200643
7
class

order
7
171549

family
7
815

genus
7
816

457390
3

SRS017103\_contig\_number\_29746SRS017307\_contig\_number\_15910SRS142980\_contig\_number\_5422
species

2302925
1

SRS014923\_contig\_number\_29628
species

species

SRS077589\_contig\_number\_420
1
821

28116

SRS017622\_contig\_number\_136
species
1

818

SRS045645\_contig\_number\_2978
species
1
